# Recent Advances on Ultrasound Contrast Agents for Blood-Brain Barrier Opening with Focused Ultrasound

**DOI:** 10.3390/pharmaceutics12111125

**Published:** 2020-11-21

**Authors:** Ambre Dauba, Anthony Delalande, Hermes A. S. Kamimura, Allegra Conti, Benoit Larrat, Nicolas Tsapis, Anthony Novell

**Affiliations:** 1 Université Paris-Saclay, CEA, CNRS, Inserm, BioMaps, Service Hospitalier Frédéric Joliot, 91401 Orsay, France; ambre.dauba@universite-paris-saclay.fr; 2 Centre de Biophysique Moléculaire and Université d’Orléans, CNRS-UPR 4301, 45071 Orléans, France; anthony.delalande@cnrs.fr; 3 Department of Biomedical Engineering, Columbia University, New York, NY 10032, USA; kamimura.hermes@columbia.edu; 4 Department of Biomedicine and Prevention, University of Rome Tor Vergata, 00133 Rome, Italy; allegra.conti@uniroma2.it; 5 Université Paris-Saclay, CEA, CNRS, Baobab, NeuroSpin, 91191 Gif-sur-Yvette, France; benoit.larrat@cea.fr; 6 Université Paris-Saclay, CNRS, Institut Galien Paris-Saclay, 92296 Châtenay-Malabry, France; nicolas.tsapis@universite-paris-saclay.fr

**Keywords:** blood-brain barrier, bubble, droplet, phase-change contrast agent, ultrasound

## Abstract

The blood-brain barrier is the primary obstacle to efficient intracerebral drug delivery. Focused ultrasound, in conjunction with microbubbles, is a targeted and non-invasive way to disrupt the blood-brain barrier. Many commercially available ultrasound contrast agents and agents specifically designed for therapeutic purposes have been investigated in ultrasound-mediated blood-brain barrier opening studies. The new generation of sono-sensitive agents, such as liquid-core droplets, can also potentially disrupt the blood-brain barrier after their ultrasound-induced vaporization. In this review, we describe the different compositions of agents used for ultrasound-mediated blood-brain barrier opening in recent studies, and we discuss the challenges of the past five years related to the optimal formulation of agents.

## 1. Introduction

The brain homeostasis is maintained by the blood-brain barrier (BBB), composed of tight junctions between endothelial cells on the vessel walls. The BBB, while preventing the entry of potentially harmful compounds, is the primary obstacle to efficient intracerebral delivery of almost all pharmaceuticals developed to treat neurological diseases, especially large molecule compounds [1]. Of the several techniques to deliver drugs across the BBB [1], the use of focused ultrasound (FUS) in conjunction with microbubbles is of great interest as it is targeted, transient, non-invasive, and safe [2]. This effectiveness of this technique was demonstrated for the first time by Hynynen et al. in 2001 [3]. After a few hours, gradual closure of the BBB and normal functioning was observed [1,4,5]. The safety has also been demonstrated in small animals and in non-human primates through histological evaluation and behavioral studies after FUS-mediated BBB disruption at multiple times and locations [6,7]. More recently, phase I and II clinical trials have shown the safety of this technique in humans [8]. This evidence strongly supports that, using suitable parameters, FUS is safe for BBB opening with a great potential to treat many brain diseases.

FUS-induced BBB opening enhances the delivery of drugs in the central nervous system [9,10,11]. Currently, the FUS-induced delivery of several compounds is under investigation for the treatment of diseases such as glioblastoma [12], neurodegenerative diseases like Alzheimer’s [13], Parkinson’s [14], or genetic diseases [15]. Additionally, FUS combined with microbubbles can achieve therapeutic results alone: it can induce, for example, neurogenesis [16] or reduce the amyloid load in Alzheimer’s disease [17]. The approach is usually combined with magnetic resonance imaging (MRI), which enables the treatment guidance, the evaluation of BBB disruption using MR contrast agents, and the monitoring of potential damages during the procedure [3,18,19,20,21,22].

**Figure 1:**
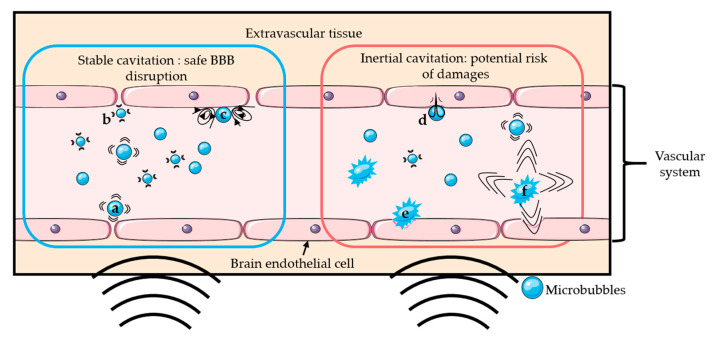
Schematic representation of several mechanisms of BBB disruption: Stable cavitation induces push- (**a**) and-pull (**b**) mechanism and microstreaming (**c**), which can permeabilize the blood-brain barrier safely. Inertial cavitation induces micro-jetting (**d**), fragmentation (**e**), and shock-wave (**f**) that can permeabilize the BBB with risks of damages.

The addition of microbubbles has reduced the amount of ultrasound energy required to open the BBB by 100-fold [2,3]. Upon sonication, microbubbles start oscillating at the frequency of ultrasound. Above a certain acoustic pressure, the previously symmetrical oscillations of the bubble become unstable [23]. Those two different regimens are respectively called stable and inertial cavitation. Inertial cavitation can induce microbubble collapse accompanied by micro-jetting, fragmentation, and shock-wave formation, which may induce vascular endothelium damages [20,22,24,25]. On the other hand, the mechanical stress generated by the stable cavitation can locally and reversibly disrupt the tight junctions present in the vascular endothelial tissue, which increases the BBB permeability [26]. It is generally accepted that stable cavitation is the preferred regime for a safe BBB opening [26]. Figure 1 schematically presents the two oscillation regimens of microbubbles and their potential effects on BBB. The use of low acoustic pressure (few hundreds of kPa) ensures the safety of the technique by limiting any potential damages such as erythrocyte extravasation, hemorrhage, and necrotic damage [27] resulting from local thermal [28] or mechanical effects [24,27].

**Figure 2:**
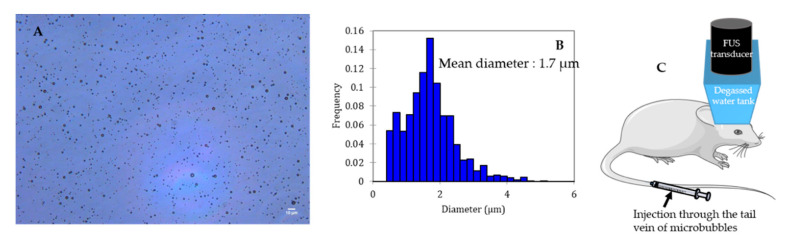
Size distribution characterization of a lipid shelled microbubble solution. Shell composition: 1,2-distearoyl-sn-glycero-3-phosphocholine (DSPC) 9:1 1,2-dimyristoyl-sn-glycero-3-phosphoethanolamine-*N*-[methoxy(polyethylene glycol)-2000] (DSPE-PEG2000). Core composition: decafluorobutane. (**A**) Microscopic picture of the solution (dilution: 1:10; scale: 10 µm), (**B**) size distribution of the solution, (**C**) schematic representation of BBB opening on mice.

Microbubbles consist of a gas core coated/encapsulated by a stabilizing shell. The coating provides a gas diffusion barrier while the gas core, composed of a heavy molecular weight inert gas, improves the bubble half-life after injection thanks to its low solubility in the surrounding medium [29,30]. Figure 2 shows a typical microscopic picture of polydisperse lipid-shelled microbubbles and their size distribution. The typical size of a microbubble is between 1 and 10 µm in diameter [29]. Sub-micronic bubbles (between 100 nm and 1 µm) are usually named nanobubbles in the literature. In order to be used for BBB opening, bubbles have to be (i) compressible to undergo cavitation, (ii) stable to circulate long enough to fulfill their duty, and (iii) non-toxic. After injection into the bloodstream, microbubbles circulate for only a few minutes before being cleared [31].

Ultrasound contrast agents (UCAs) initially designed as echogenic contrast agents and clinically approved for diagnostic applications have also been shown capable of disrupting the BBB. However, bubbles can be formulated specifically for BBB disruption. Additionally, UCAs can be loaded with MRI contrast agent molecules, which allows imaging of their biodistribution. Targeting ligands can be added to the UCAs surface to increase their specificity or engineered with embedded drugs for targeted release, which reduces systemic drug effects.

An ultrasound can be used to convert droplets, called emulsions or phase-change contrast agents (PCCA), into microbubbles. For this reason, a droplet can also be designed for BBB opening. In vivo, a sufficient peak rarefactional pressure is necessary to vaporize the droplet’s liquid core [32]. Acoustic droplet vaporization (ADV) and the cavitation of the resulting bubble can induce BBB opening [33].

A systematic review of the recent literature was performed to build a database of the different materials employed for the shell and core of agents used to open the BBB with FUS. A discussion about the different possibilities to find the right balance between reaching a stable cavitation regimen and agent stability is presented. Following that, we present a summary of the different multifunctional agents used to disrupt the BBB with FUS, enhance MRI contrast, or molecularly target a specific area of the brain or/and carry drug all at once. We finish the review with a description of the latest developments on engineered biomolecules, which open new avenues for cell-based therapy and diagnostic. To the best of our knowledge, this is the first review to provide a database of the different materials recently used to disrupt the BBB transiently with FUS.

## 2. Recent Advances on Sono-Sensitive Agents for Ultrasound-Assisted Blood-Brain Barrier Opening

### 2.1. Method of Literature Search

We searched on PubMed in October 2020 for all studies on BBB disruption induced by FUS in conjunction with microbubbles or nanodroplets using search terms (microbubble OR nanodroplet OR nanoemulsion OR “phase-change contrast agent”) AND (“blood-brain barrier” OR “brain-targeted drug delivery”) AND (ultrasound). We limited the search to the last 5 years (from 7 May 2015 to 1 October 2020), which yielded 247 studies. After screening titles and abstracts, we only kept articles that studied FUS-induced BBB opening in vivo as a therapeutic purpose. We thus excluded studies about BBB inflammatory response, passive acoustic mapping, and computer simulation. We also excluded studies that did not provide enough information about the agent used to disrupt the BBB.

**Figure 3:**
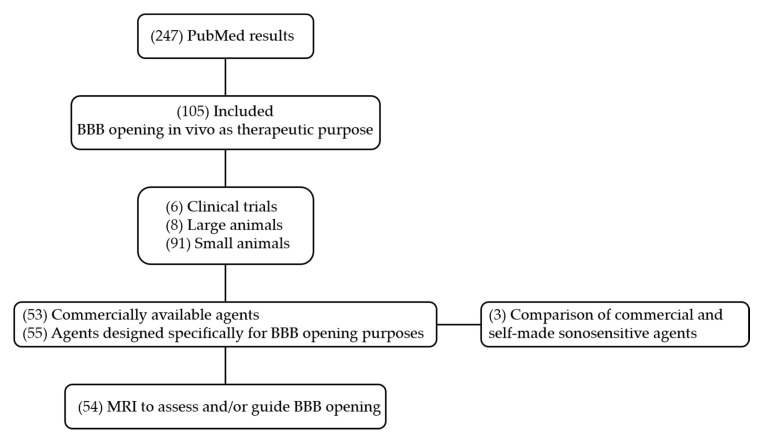
Flow chart of PubMed.

As a result, 105 studies were included for data extraction and synthesis. The selection included 6 clinical trials, 8 preclinical studies on large animals, and 91 on small animals (rodents and rabbits). 53 studies used commercially available contrast agents, and 55 studies described agents explicitly designed for BBB opening purpose. Three of these studies compared commercial and self-made contrast agents. Among these studies, 54 used MRI to assess and/or guide BBB opening. The flow diagram of the search is presented in Figure 3.

This search has brought to our attention several reviews that supplement the present one [31,34]. In addition, an overview of other strategies for brain drug delivery can be found in [35].

### 2.2. Commercial Ultrasound Contrast Agents

Commercially available UCAs used for BBB opening are either clinically approved as imaging contrast agents (Food and Drug Administration or European Medicines Agency) or are being developed specifically for therapeutic purposes. The clinically approved microbubbles used to disrupt the BBB with FUS are Definity^TM^ (Lantheus Medical Imaging, North Billerica, MA, USA), SonoVue^®^/Lumason^®^ (Bracco, Milan, Italy), Optison^TM^ (GE Healthcare, Milwaukee, WI, USA), and Sonazoid^®^ (GE Healthcare, Milwaukee, WI, USA). Other commercially available UCAs resulted in successful BBB opening such as USphere^®^ (Trust Bio-sonics, Zhubei City, Taiwan), SIMB^®^ (Advanced Microbubbles Inc, Boulder, CO, USA), Vevo MicroMarker^®^ (Fujifilm, Toronto, ON, Canada), BR-38^®^ and others (Bracco Suisse SA, Geneva, Switzerland), and bubbles ordered from Targeson Inc. (San Diego, CA, USA) These agents consist of lipid or protein shells with gaseous sulfur hexafluoride (SF_6_) or perfluorocarbon (PFC) cores. Table 1 summarizes the commercial and non-commercial UCAs used to disrupt the BBB with ultrasounds in pre-clinical studies during the past 5 years, their composition, and associated references.

We came across four studies comparing different commercially available bubbles on their abilities to open the BBB by assessing Evans blue leakage in the brain (commonly used dye for BBB permeability assessment, otherwise blocked by the BBB). The obtained results for these 4 studies are summarized in Table 2, along with the bubble type, injection dose, and ultrasonic parameters used for the BBB disruption. Briefly, Shin et al. compared a dose of SonoVue at 30 µL/kg with two different doses of Definity at 20 µL/kg and 100 µL/kg. Definity microbubbles at a 20 µL/kg dose were more effective for BBB opening and led to fewer damages than SonoVue. For the 100 µL/kg dose of Definity, the BBB opening was more important, and the level of tissue damages (histological evaluation) was similar to SonoVue microbubbles at 30 µL/kg [36]. Wu et al. compared SonoVue, Definity, and USphere at an injected microbubble concentration of 4 × 10^7^ bubbles/kg. For a given set of sonication parameters, the order of Evans blue penetration (from the most important to the weakest) was: SonoVue, Definity, Usphere [37]. Bing et al. compared Optison and Definity performances. The concentration of bubbles was adjusted to inject the same gas volume (1.1–1.2 µL/mL). Evans blue leakage was more important for Optison microbubbles than for Definity [38]. Finally, Omata et al. compared SonoVue and Sonazoid at an injected microbubble concentration of 3 × 10^9^ bubbles/kg and showed a higher Evans blue leakage with the Sonazoid bubbles [68].

In the Wu et al. study, a 200 µL/kg dose of SonoVue microbubbles successfully opened the BBB without damages using a higher peak negative pressure and a more prolonged exposure than the Shin et al. study where damages on the rat brain were observed for a 20 µL/kg dose of SonoVue. This inconsistency between the two studies points out that the BBB opening effectiveness and safety should be studied in-depth, ideally comparing different commercially available bubbles. Omata et al. showed that a 30,000 µL/kg dose of SonoVue successfully opened the mice BBB without damages. However, the comparison with Wu et al. and Shin et al. is trickier as they studied mice for which the skull is thinner than rats. Furthermore, ultrasound parameters such as the frequency used (3 MHz) for BBB opening differs from other studies making the comparison challenging. As another example, the Wu et al. study demonstrates a higher efficacy of SonoVue over Definity while the Shin et al. study tends to the opposite direction. Importantly, injected doses play a critical role. Besides, commercially available agents explicitly made for BBB opening purposes would be desirable.

### 2.3. Design of Specific Agents for Blood-Brain Barrier Opening

While clinically-approved contrast agents expedite therapeutic clinical trials, their compositions originally developed for diagnostic imaging can be suboptimal for BBB opening [127]. Moreover, designing specific BBB disruption agents makes possible their use as multifunctional agents for imaging and drug delivery purposes in theranostics.

Agents designed for BBB disruption, like UCAs, have to fulfill several criteria such as (i) easily reachable stable cavitation regimen, (ii) long in vivo circulation stability, and (iii) storage stability. Thereby, bubbles must undergo sufficient oscillations for a relatively low peak rarefactional pressure before reaching inertial cavitation. Stable and inertial cavitation thresholds have to be assessed, meaning that the lowest acoustic pressure required for the bubble to reach these regimens has to be estimated. The vaporization threshold for droplets, namely the magnitude of acoustic pressure required to convert a liquid droplet into a gaseous bubble, also has to be considered [128]. Over the 55 studies describing agents designed for BBB opening procedures, only 15 performed at least one test to assess cavitation thresholds or stability performances (Table 3). These tests are crucial for the success and safety of the experiments [91]. Therefore, they should be performed on a routine basis, especially since those parameters can be incredibly different from an agent to another (acoustic stability on echography images ranging from few seconds [68] to days [88] depending on the bubble). Currently, most of the bubble-based therapeutic ultrasound protocols are limited to a few minutes or require a repeatable injection of fresh UCAs to extend the circulation of microbubbles in the body and increase the procedure’s efficiency. For commercial microbubbles, the half-life in the bloodstream is typically lower than 10 min [129], and extending the in vivo circulation of these agents is desirable.

**Figure 4:**
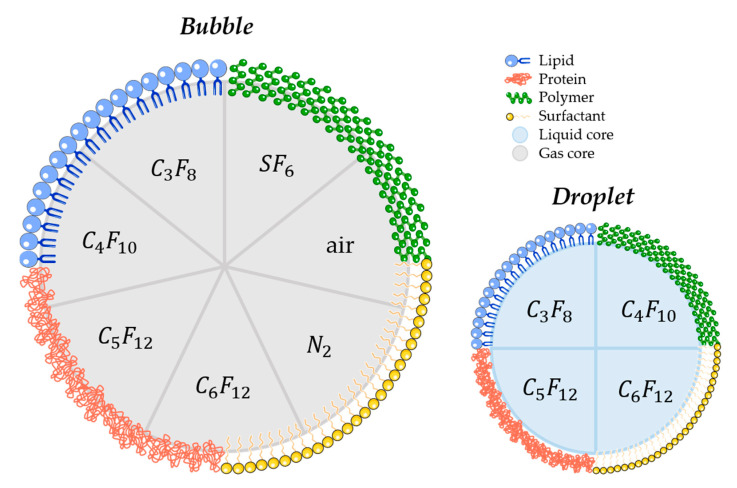
Schematic illustration of most commonly used materials for shell and core composition of agents used for FUS-induced BBB. (C_3_F_8_ octafluoropropane; C_4_F_10_: decafluorobutane; C_5_F_12_: dodecafluoropentane; C_6_F_12_: perfluoromethylcyclopentane; SF_6_: sulphur hexafluoride; N_2_: nitrogen).

Several parameters may influence the bubbles/droplets circulation time as well as the cavitation threshold. For this reason, aspects such as size, chemical composition, shell properties (such as surface tension, elasticity, thickness, surface chemistry), and core properties (molecular weight, density, and boiling point for droplets) have to be taken into account when designing a new agent for FUS-induced BBB opening purpose [127,128,129]. Concretely, air, nitrogen, and mostly PFC and SF_6_ are used as the core gases, while surfactants, lipids, proteins, polymers, or a combination of these materials are used for the shell. For that matter, Figure 4 schematically represents the different possibilities of BBB disruption agent composition.

#### 2.3.1. Bubble or Droplet?

The most commonly used agents for BBB opening with FUS are lipid-shelled microbubbles. PCCA is an attractive alternative to conventional microbubbles. Apart from the core phase, PCCA composition is very similar to that of bubbles [31]. Moreover, stable droplets can be generated through the condensation of commercial PFC bubbles [130]. Like their gas-core counterparts, PCCA is nontoxic at low doses [131]. The shell’s surface tension creates a pressure difference between the internal and external environment, known as the Laplace pressure. This pressure allows the compounds to remain stable in the bloodstream [128] until reaching vaporization conditions (i.e., increased temperature and rarefactional acoustic pressure) [131,132].

PCCA presents a significantly longer circulation time in vivo than microbubbles since the liquid core prevents gas dissolution [128]: depending on the choice of PFC, droplets may persist stably for hours in vivo [133]. Due to their smaller size, PCCA can also potentially extravasate in the leaky vasculature within tumors, unlike microbubbles [31,80,133].

The ultrasound frequency can be tuned to reduce the pressure amplitude required for droplet vaporization (ADV) [134]. Concretely, the vaporization threshold decreases with increasing signal frequency, meaning that a large number of droplets will be activated using high excitation frequency. Conversely, the use of high frequency will increase the shell rupture threshold of bubbles (resulting in inertial cavitation) [135], decreasing the risk of potential damages. Therefore, increasing the frequency looks promising for the safe application of UCA-mediated therapeutic ultrasound. However, FUS-induced BBB opening is usually performed at low frequency (from 0.2 to 3 MHz) to reduce the ultrasound beam’s attenuation through the skull. Another option to reduce the PCCA vaporization threshold would consist of the formulation of low-boiling point droplets at the stability’s expense, as described in the following section [131].

**Figure 5:**
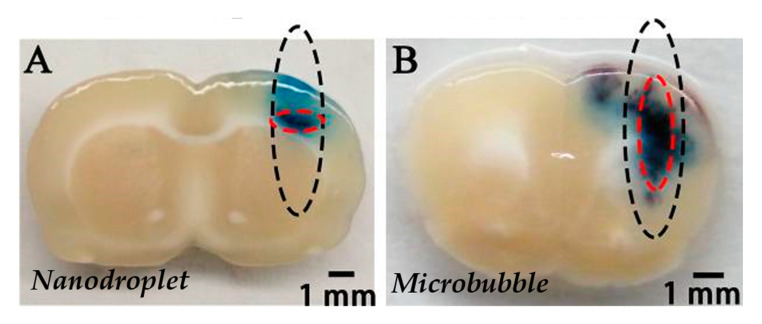
Distribution pattern of Evans blue extravasation when PEG-PLGA-C_5_F_12_ nanodroplets (**A**) or lipid microbubbles (**B**) were used for BBB opening with FUS on male Sprague-Dawley rats. The black dotted circles show the half-maximum of the pressure amplitude of the focal zone. The red dotted circles show the deep blue region of EB extravasation, which indicates the location of the BBB opening. Extracted from Zhang et al. [80], Impact Journals, 2017.

In our literature search, the BBB disruption with PCCA has been achieved only three times [33,80,133]. Chen et al. firstly demonstrated BBB disruption with lipid-shelled and C_4_F_10_ core PCCA and successfully delivered 3 kDa dextran to C57BL/6 mice brain without damages. Interestingly, they showed that PCCA had a higher BBB opening pressure threshold (0.450 MPa vs. 0.225 MPa) than the same material microbubbles [133]. In the first study using polymeric PCCA (PEGylated-PLGA shell and C_5_F_12_ core) for BBB opening, Zhang et al. demonstrated that their PCCA has better-focusing abilities than lipid shelled microbubbles (distribution pattern of Evans Blue extravasation on Figure 5) [80], thus reducing side effects outside the focal zone [32,80]. More recently, Wu et al. successfully delivered 40 kDa dextran to the C57BL/6 mice brain without damage with lipid shelled PCCA [33].

Recently, “acoustic clusters,” produced by electrostatic complexation between negatively charged bubbles and positively charged droplets, have been evaluated for BBB disruption [79]. Indeed, Åslund et al. demonstrated that acoustic clusters made from Sonazoid^®^ microbubbles and perfluoromethylcyclopentane microdroplets stabilized with a phospholipid membrane were able to safely and temporarily permeabilize the BBB, using low acoustic pressure and deliver a larger amount of Omniscan^TM^ (about 30% increase of the gadodiamide signal ratio between the treated part and the non-treated contralateral part of the brain) and 45 kDa molecules into the rat brain in comparison with Sonazoid^®^ microbubbles. Briefly, after intravenous injection of the clusters, ultrasound is applied, and the microbubbles transfer the acoustic energy to the attached droplets, which undergo ADV. The acoustic energy transfer enables the microdroplet’s liquid-to-vapor phase transition at low acoustic pressure (0.28 MPa in the Åslund et al. study). The resulting acoustic-cluster-bubbles undergo a rapid expansion to approximately 25 μm and transiently deposit in the local microvasculature, stopping the blood flow for up to 10 min. This activated bubble is in direct contact with the endothelial wall ensuring optimal coupling between the vessel wall and the oscillating bubble. The application of ultrasound (0.09 MPa; 1 MHz in the Åslund et al. study) increased the vasculature’s local permeability (Omniscan^TM^ signal in the treated part was 50% higher compared to the non-treated part of the brain in the Åslund et al. study). The acoustic-cluster-bubble stays for typically 10 min, prolonging the treatment time window compared to regular contrast microbubbles [79]. While this approach is innovative, it might be deleterious to stop the blood flow for up to 10 min in the sonicated area.

#### 2.3.2. Core Composition

The gas chosen for the core must be non-toxic, hydrophobic, and must have a low solubility to prevent gas leakage through the agent’s shell. Heavy gases are preferentially used because bubbles made out of them possess a longer half-life [129,136]. Sulfur hexafluoride (molecular weight, MW = 146.06 g/mol), air (MW = 28.98 g/mol), octafluoropropane (MW = 188.02 g/mol), dodecafluoropentane (MW = 288.03 g/mol), or decafluorobutane (MW = 238.03 g/mol) are often used since their biocompatibility is reported [137]. The PFC must be selected considering the ultrasound pressure needed to induce safe BBB disruption, and in the case of PCCA, the ADV threshold [138]. Acoustic pressure consideration for vaporization or cavitation threshold is crucial for large animal and human applications, as the transmission of high acoustic pressure through the skull remains challenging and requires the use of very sophisticated and expensive ultrasound devices [127].

The Laplace pressure, defined as the differential pressure between the outside and the inside of the droplet, prevents the spontaneous vaporization of the PFC droplets in vivo.

$$\Delta P=Pinside-Poutside=\;\frac{2\gamma }{r}$$

where *γ* is the surface tension, and *r* is the bubble/droplet radius. The hydrophobicity of liquid PFC leads to relatively high surface tension, thereby increasing the boiling point. After ADV, the droplet becomes a larger bubble, which remains stable due to the low solubility of the PFC [128]. Thus, depending on the boiling point of the selected materials, droplets can vaporize at different acoustic pressures; the use of PFC with lower boiling points results in easier vaporization at lower acoustic pressures [32].

Omata et al. developed several gas-loaded microbubbles with identical shell compositions and assessed their stability and the effects of the encapsulated gas on BBB opening and drug delivery to the brain. They have also compared those self-made microbubbles to commercial ones (Sonazoid and SonoVue) on the same specifications [68]. The main results of this study are presented in Table 4. The in vitro half-life results suggest that the rank order of gas-bubble stability in vitro, from the longest to the shortest, is C_4_F_10_, C_3_F_8_, and SF_6_. The in vivo experiments were performed on mice kidneys and suggested the same tendency, except for the Sonazoid bubbles that appear to be less stable in the circulation despite their high stability in vitro. Omata et al. suggest that this is due to Sonazoid shell composition, which contains hydrogenated egg phosphatidylserine, a lipid known to be rapidly uptake by some liver cells. On the BBB opening and drug delivery assessments, C_3_F_8_ and C_4_F_10_ cores continue to show the best performances. Moreover, all the BBB disruption was reached without damages. Sonazoid resulted in high Evans blue delivery despite its short in vivo half-life. For this reason, Omata et al. repeated the BBB opening assessment for Sonazoid and C_3_F_8_ and C_4_F_10_ self-made bubbles but applying ultrasound 3 min after injection. Under these conditions, Evans blue leakage was still observed on the brain’s sonicated part, but the difference with the untreated part of the brain was not significant for the Sonazoid bubbles. The study concluded that self-made C_3_F_8_ and C_4_F_10_ were more stable and efficient for BBB opening and drug delivery.

Concerning the PCCA core, Wu et al. compared two low-boiling point PCCA for their abilities to disrupt the BBB and deliver dextran molecules to the murine brain. Octafluoropropane (boiling point −36.7 °C) PCCA successfully delivered 40-kDa dextran to the brain at 300 kPa and 450 kPa without evidence of cavitation damage. Using decafluorobutane (boiling point −1.7 °C) PCCA, the successful delivery of dextran at 900 kPa was associated with tissue damage due to inertial cavitation. Interestingly, no delivery was detected at a lower pressure of 750 kPa [33]. No study reported PCCA made with a sulfur hexafluoride core (boiling point is -68.25 °C). Those PCCA would probably not be stable enough to be injected in vivo. Those studies’ results are very informative and suggest that the comparison of core compositions repercussions on stability, acoustic responsiveness, and BBB opening ability should be further investigated.

#### 2.3.3. Shell Composition

A proper shell must balance flexibility and sturdiness: it has to be flexible enough to let the bubble oscillate at relatively low acoustic pressure while having a proper surface tension to prevent gas leakage from the core or keep it in its liquid form at physiological temperature for droplets.

The most elastic shells are made of phospholipids, while the stiffer ones are made of polymers or proteins [30]. Lipid-shelled microbubbles are highly responsive to ultrasound, while the polymer and protein-shelled microbubbles are very stable and require higher acoustic pressure to undergo cavitation [24,139]. PEG-surfactants are systematically used with phospholipid shells due to their ability to appreciably lower surface tension [102]. Other surfactant shells have also been explored in echogenic contrast agent fabrication [128].

Wu et al. examined the effects of lipid acyl chain lengths (C16, C18, C24) for molecular delivery to the murine brain after BBB disruption. Increasing lipid tail length and its shell rigidity resulted in a significant increase in the delivery of 40-kDa dextran. The difference in molecular delivery between the different shells was weaker with higher pressure [92].

Pouliopoulos et al. compared different lipid shell microbubbles shelled on their acoustic stability in vitro [102]. The shells were composed of 1,2-distearoyl-sn-glycero-3-phosphocholine (DSPC) and 1,2-distearoyl-sn-glycero-3-phosphoethanolamine-*N*-[methoxy(polyethylene glycol)2000] (DSPE-PEG2000) with different molar ratio (6:1; 9:1; and 12:1). The bubbles were excited with a 0.5 MHz FUS transducer, and passive cavitation detection was performed with a 7.5 MHz single element FUS transducer. The energy transmitted by the excited bubble was processed to appraise the different shell acoustic stabilities. Eventually, a 9:1 ratio was found to provide higher acoustic stability [102]. DSPC:DSPE-PEG2000 at a molar ratio of 9:1 is the most commonly formulated lipid shell for microbubble self-made for BBB disruption.

The electrical charge of the shell can also influence its properties. Cationic bubbles have been explored for BBB opening [104,105,106] due to their ability to form electrostatic complexes with negatively charged molecules such as nucleic acids (DNA, RNA, oligonucleotides) [30]. Besides, cationic bubbles have shown some advantages compared to neutral bubbles. Even though it might be cleared by the organism faster than neutral bubbles [30], it can conversely present better stability below 37 °C when the cationic lipid used for the shell has a higher transition temperature than the neutral one [107]. Tan et al. compared three lipid bubbles with different surface charges (neutral: Neu, slightly cationic: Scat, and cationic: Cat) with neutrally charged Definity microbubbles in vitro by performing shell elastic modulus and turbidity measurements and by evaluating their ability to increase the permeability of epithelial brain cells [39]. The different surface charges were obtained by varying the lipid compositions and the chemical termini of the lipid shell. Neu bubbles were formulated at a 9:1 molar ratio of DSPC:DSPE-PEG2000, Scat bubbles were formulated at a 9:1 molar ratio of DSPC and 1,2-distearoyl-sn-glycero-3-phosphoethanolamine-*N*-[amino(polyethylene glycol)2000] (DSPE-PEG2000-Amine), and Cat bubbles were formulated at a 9:2:1 molar ratio of DSPC, DSPE-PEG2000-Amine, and 1,2-distearoyl-3-trimethylammonium-propane (DSTAP). All the bubbles were about the same size (around 1 µm), and the average Zeta potential measured was −10.6 ± 0.8 mV, 17.0 ± 0.3 mV, 29.8 ± 0.3 mV, −1.1 ± 0.2 mV for the Neu, Scat, Cat, and Definity bubbles, respectively. The shell elastic modulus was higher for Definity bubbles and similar for the three homemade bubbles suggesting that (i) Definity bubbles have a higher cavitation threshold than the homemade agents and (ii) the bubble surface charge does not influence its elasticity. Turbidity measurements showed that after 5 min sonication (*fc =* 3 MHz; MI = 0.8), the number of bubbles remaining in the water bath was lower for SCat bubbles (20%) than for the others (Neu: 40%; Cat: 80%; Definity: ~100%) suggesting that the SCat bubbles have the lowest inertial cavitation threshold as they were more deteriorated than the other type of bubbles. Finally, cell permeability to 70-kDa dextran assessment showed best results for the Scat bubbles which might be explained by their lower cavitation threshold and electrostatic interactions between their cationic shell and the negatively charged cell membranes. Based on these results, SCat bubbles were evaluated for BBB disruption in vivo.

Once again, we believe that shell composition repercussions on stability, acoustic responsiveness, and BBB opening performances must be further studied.

#### 2.3.4. Fabrication Methods

The agent used for BBB disruption can be made with several methods. The three most common bubbles fabrication methods are sonication, shaking, and microfluidics [140]. Sonication is performed with a high-frequency vibration (typically 20 kHz) horn tip at the gas/water interface. Shaking is another mechanical agitation process consisting of a device vibrating along the long axis of a vial at 4000 Hz. This method is used to generate Definity^®^ microbubbles, for example. Sonication and shaking produce polydisperse microbubbles very rapidly and economically. If a monodisperse distribution of the bubble solution is desired, the solution can be centrifugally sorted at the yield’s expense. Moreover, sonication can produce larger amounts of bubbles than shaking. Microfluidic methods, which have been more recently proposed, are interesting because they produce monodisperse microbubbles and offer directed assembly for more sophisticated structures. However, those methods have relatively slow rates, and they are difficult and expensive to employ.

Sheeran et al. reported the following techniques for the fabrication of phase-change PFC droplets: shaking, sonication, extrusion, condensation, and microfluidic techniques [141]. As for bubble fabrication, shaking methods can achieve micro-scale emulsions simply and rapidly but produce a broad size distribution and have low reproducibility between batches. In contrast, sonication techniques can produce nano-scale droplets with a narrower size distribution but may require size exclusion processes such as filtration. Microfluidic techniques offer a much narrower size distribution, but the ease and speed of manufacture may be limited [128]. Among the PCCA used for BBB disruption, microbubble condensation [33,133], and double sonication method [80] have been used. The condensation method requires to decrease the ambient temperature and raise the ambient pressure of the microbubble suspension. This technique enables the simple production of high-concentration PCCA and offers the possibility of modifying and manipulating microbubbles at the microscale prior to condensation. However, this method produces polydisperse droplets if the preceding microbubble suspension is highly polydisperse (however, differential centrifugation can be performed to overcome this) [141]. The fabrication method is essential as it impacts the agent size distribution and concentration of the suspension.

#### 2.3.5. Size Distribution and Concentration

Concentration and bubble size influence their dissolution and clearance rate [127]. Indeed, as Borden et al. described, the lipid monolayer bubble’s dissolution rate increases with decreasing bubble size. Thus, smaller microbubbles dissolve faster than larger ones [140]. Sirsi et al. demonstrated that increasing bubble diameter and/or concentration increases in vivo circulation persistence by comparing bubbles of different sizes (1–2 µm; 4–5 µm and 6–8 µm) and at different concentrations (between 10^7^ and 10^9^ bubbles/mL) [142]. However, for intravenous administration, the microbubble diameter must be smaller than 6 µm [30]. Cheng et al. also studied submicronic bubbles (288 ± 17.6 nm) stability regarding the concentration and observed that the in vitro persistence was higher with a higher concentration (concentration ranging between 10^7^ and 10^11^ bubbles/mL) [108].

While the droplet vaporization threshold is inversely proportional to droplet diameter [128], it has also been demonstrated that bubble cavitation behavior depends on its size. Indeed, smaller bubbles were more susceptible to destruction by fragmentation under acoustic stimulation [143]. Moreover, Choi et al. showed that increasing the bubble diameter decreased the cavitation thresholds (0.30 and 0.46 MPa for 1–2 µm bubbles and 0.15 and 0.30 MPa for 4–5 µm bubbles) and that BBB opening is higher with larger diameter bubbles for every pressure tested [144]. However, Bing et al. have observed in vivo ultra-harmonic emission at lower acoustic pressure for submicronic bubbles (288 ± 17.6 nm; 0.23 MPa) in comparison to Definity and Optison microbubbles (~2 µm; 0.34 MPa and 0.42 MPa respectively), suggesting that BBB opening might be achieved with the high activity of submicronic bubbles under relative lower focal pressures. The authors noted that this observation might differ from Choi et al. study due to the relatively high concentration of the submicronic bubbles (737 µL/kg). As a result, part of the bubbles may combine and form a cluster with a larger diameter compared to both Optison and Definity [38].

### 2.4. Bimodal Ultrasound-MRI Contrast Agents for BBB Disruption

MRI is currently used to guide and evaluate the efficiency of therapeutic ultrasound procedures. It allows precise targeting, identification of the lesion, and dynamic feedback on the extent of BBB disruption via MR contrast leakage [1,18]. Two types of agents are used to enhance the contrast on MR images: (i) paramagnetic agents, which consist of a chelate with a paramagnetic core (usually gadolinium) or (ii) superparamagnetic agents composed of iron oxide nanoparticles coated with a hydrophilic organic protective layer such as dextran [145].

Most of the studies reported the use of paramagnetic agents made of gadolinium chelates commercially available such as Gadovist^®^ (Gd-DO3A-butrol), Dotarem^®^ (Gd-DOTA), Magnevist^®^ (Gd-DTPA), Omniscan^®^ (Gd-DTPA-BMA), and Multihance^®^ (Gd-BOPTA) that are co-administered with the UCAs. Aryal et al. incorporated a gadolinium-labeled lipid in the lipid bilayer of liposomes injected as an MRI contrast agent. After BBB opening on mice with Optison^®^ microbubbles, the signal intensity was slightly higher on longitudinal relaxation time (T1)-weighted images for the sonicated hemisphere than the control volume, indicating that the gadolinium-labeled liposomes were effectively delivered to the brain and visible on MR images. Two different sizes of liposomes were compared (77.5 nm and 140 nm), and the relative increase in MRI signal intensity was greater for smaller liposomes than larger ones [70].

Conventional UCAs have also been used for MRI [146] due to the gas-liquid interface producing large local magnetic susceptibility differences visible in transversal relaxation time (T2)-weighted MR images. For that matter, Cheung et al. used two types of bubbles (custom-made air-filled and albumin-coated microbubbles and SonoVue^®^ microbubbles) for in vivo dynamic brain MRI in Sprague-Dawley rats. Transverse relaxation rate enhancements were observed in the brain after bubbles intravenous injection [147].

**Figure 6:**
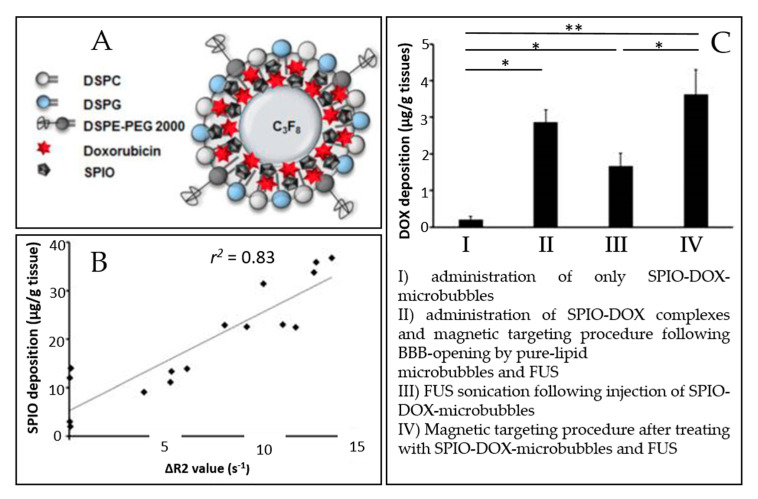
An example of a multimodal agent for BBB disruption, MRI, and drug delivery purposes (extracted from Fan et al. [109], Ivyspring International Publisher, 2016). (**A**) Illustration of SPIO-DOX-microbubble structure. (**B**) Correlation between SPIO deposition and ΔR2 value. (**C**) DOX accumulation measured by high-performance liquid chromatography. *: *p* < 0.05; **: *p* < 0.01.

The microbubble response can be further enhanced by incorporating paramagnetic or superparamagnetic particles into their shells, giving rise to multimodal contrast agents used for BBB disruption and dynamic contrast-enhanced MRI. Liao et al. reported the use of perfluorocarbon-filled albumin-(Gd-DTPA) microbubbles for monitoring FUS-induced BBB opening [148]. T1-weighted MRI confirmed the BBB disruption, and T2-weighted MRI allowed to detect intracerebral hemorrhage. Besides, Fan et al. formulated a multimodal, therapeutic, and active-targeting microbubble encapsulating a superparamagnetic iron oxide-doxorubicin (SPIO-DOX) complex. The lipid microbubble filled with perfluoropropane successfully opened the BBB upon sonication, and the magnetic activation of SPIO nanoparticles triggered the release of chemotherapeutic agent DOX into rat glioma. Figure 6 schematically represents the SPIO-DOX-microbubble complex, the SPIO deposition as a function of 1/T2 values in MR images, and the DOX deposition into the rat brain as a function of the treatment used. The SPIO deposition into the rat brain tumor was correlated with differences in 1/T2 values in MR images (*r*^2^ = 0.83) and with DOX deposition (*r*^2^ = 0.79), supporting the theranostic capabilities of the SPIO-DOX-microbubble complex [109].

PFC droplets can theoretically be observed on ^19^-F MRI since it has been reported in vitro [149]. Nevertheless, in vivo ^19^-F MRI imaging remains challenging due to the low PFC quantities available in the brain after BBB disruption.

### 2.5. Drug Delivery and Targeting

This section is focused on microbubble-assisted drug delivery and targeting strategies to the brain recently exploited. Exhaustive reviews already detail the different possibilities for drug delivery to the brain in conjunction with FUS [34,150]. Some of them are specifically dedicated to cancer treatment [151,152] or gene delivery and targeting [30].

#### 2.5.1. Targeting

Localized BBB opening can be achieved using physical stimuli (such as FUS or magnetic field), while functionalized agents with a ligand can target overexpressed receptors associated with the treated pathology.

Fan et al. used magnetic targeting as a physical targeting option: particles can be magnetized and become physically sensitive to external magnetic fields. This approach was validated in vivo using the SPIO-DOX-microbubble complex described earlier (schematically represented in Figure 6A). It increased SPIO deposition in the rat brain by 2.8 fold [109]. As well, Wu et al. made lipid-shelled bubbles conjugated with cationic polyethylenimine-coated super paramagnetic iron oxide particles (PSPIO) to open the BBB on mice. The SPIO, here again, was used for magnetic targeting and enhanced the BBB opening by 2.8-fold compared with unconjugated bubbles [125].

Molecular targeting of the circulating microbubbles would allow a BBB opening in the desired area without affecting healthy tissues. Typical ligands used for molecular targeting are antibodies or peptides. Table 5 summarizes the different molecular targeting and drug/gene-complexed bubble options for BBB opening and therapeutic purposes found in the recent literature.

Chen et al. developed des-octanoyl ghrelin-conjugated microbubbles loaded with TGFβ1 inhibitor to disrupt BBB on glioma-bearing mice. Des-octanoyl ghrelin is a ligand that can bind with BBB. Authors have observed higher BBB disruption with the des-octanoyl ghrelin-conjugated microbubbles than the unconjugated ones (negative contrast intensity of superparamagnetic iron oxide nanoparticles on the T2-weighted MRI images was 0.81-fold higher for the conjugated microbubbles) [88].

Specific biomarkers of the blood-tumor barrier (BTB) could be used to target microbubbles toward these tumors. Indeed, gliomas and brain metastases are tumors known to compromise the integrity of the BBB, resulting in a vasculature known as the BTB, which is highly heterogeneous and characterized by numerous distinct features, including non-uniform permeability and active efflux of molecules [153]. Vascular endothelial growth factor receptor 2 (VEGFR2) is one of the selected targets as it is a specific endothelial molecular marker of angiogenesis, which is exceptionally high in tumor growth and, thus, overexpressed in BTB [154]. Moreover, the inhibition of VEGFR2 with antibodies results in prolonged survival in cancer patients [154]. Functionalized bubbles/droplets with VEGFR2-targeted ligand were formulated for BTB targeting. Chang et al. have used anti-VEGFR2 antibody-conjugated cationic microbubbles to target VEGFR2 in the rat BTB. The microbubble targeting efficiency was evaluated in vitro on C6 glioma cells and was 99.4 ± 0.3%, while it was 6.4 ± 1.2% for unconjugated microbubbles [104]. Most of the targets studied, such as VEGFR2, are directed to endothelial cells. However, it is also possible to target receptors directly expressed on the malignant cells, i.e., folate receptors. Hence, Fan et al. used this receptor as a target for BBB opening and gene delivery in the C6 glioma rat model. They have demonstrated the targeting ability on C6 glioma cells of folate-conjugated DNA-loaded cationic microbubbles in vitro: the folate increased the targeting ability of the complex by 7.6 fold [107]. Besides, Zhao et al. used Asn-Gly-Arg (NGR), a peptide motif that can be used to target CD13 receptors. CD13 is overexpressed in glioma cells and neovascular endothelial cells. In vitro, NGR-linked-*shBirc5*-loaded liposome complex linked to lipid-shelled microbubble demonstrated a better *shBirc5* gene transfection on C6 glioma cells for the targeted microbubble compared to the untargeted ones (36.25% of transfection efficiency vs. 21.26%), thereby demonstrating the targeting profits [110].

Molecular targeting was always associated with drug/gene loaded into the bubble in the browsed literature. Indeed, molecular targeting may improve drug delivery: Fan et al. have observed better gene transfection efficiency in vivo for their folate-conjugated microbubbles than those without conjugation (luciferase gene expression 4.7 fold higher after 24 h) [107].

#### 2.5.2. Drug and Gene Delivery

Drug delivery through the BBB with FUS can be reached by (i) co-injection of microbubbles and free drugs or drug carriers or (ii) encapsulating or covalently linking the therapeutics to the agent shell [32,129]. Loading drugs into the shells of microbubbles enables better spatial control to deliver the drug to the treated site. The encapsulation of the drug would also decrease the side effects induced by the circulation of a high drug dose in the vasculature [30,32].

Non-viral gene delivery by plasmid DNA complexation with bubbles was studied in numerous studies [89,104,105,107,110,111,112]. Thereby, glial cell line-derived neurotrophic factor (GDNF) [89,111,125], brain-derived neurotrophic factor (BDNF) [89] and nuclear factor E2-related factor 2 (Nrf2) [112] have been studied for Parkinson’s disease treatment. Gene therapy with neurotrophic factors is a promising approach to improving current Parkinson’s disease therapy. It has been found to reduce progressive neuronal loss and play a crucial role in the development, survival, and maintenance of the central and peripheral nervous system [89]. Nrf2 is a nuclear factor that activates the antioxidant response element pathway and protects the brain by regulating redox status. Nrf2 might be useful in Parkinson’s disease therapy as reactive oxygen species play an important role in disease development [112]. Thus, these factors have been encoded in plasmid DNA and complexed with microbubble for BBB disruption and drug delivery on Parkinson’s disease rodent model [89,111,112,125]. Neurotrophic factor delivery provided a neuroprotective effect by showing evidence of improvement of behavioral deficits [89,111,125], while Nrf2 gene transfection enabled the reduction of reactive oxygen species levels [112].

Interestingly, Wu et al. have used *pPrestin*-microbubble to disrupt the BBB, modify, and activate neurons within mice brain for spatiotemporal neuromodulation [105]. Prestin is a transmembrane protein that exists in the mammalian auditory system and functions as an electromechanical transducer. The cellular transfection rate with *pPrestin*-microbubble was 1.5-fold higher than with commercial transfection agents (LT-1) [105].

Chang et al. used anti-tumor suicide gene therapy for glioblastoma therapy on Sprague-Dawley rats using cationic VEGFR2-targeted microbubbles, complexed with luciferase gene and herpes simplex virus type 1 thymidine kinase/ganciclovir (gene suicide system) encoding plasmid DNA (*pLUC* and *pHSV-TK/GCV*). Anti-tumor suicide gene therapy involves tumor-targeted transfection of a suicide gene that encodes an enzyme for converting non-toxic prodrugs into toxic products to kill tumor cells. Both VEGFR2-targeting and *pHSV-TK* contributed to improving the anti-tumor efficiency: 25 days after tumor implantation, the tumor volume was 9.7 ± 5.2 mm^3^ for the *pHSV-TK/GCV*-loaded VEGFR2-targeted microbubbles-treated group, 40.1 ± 4.3 mm^3^ for the untargeted *pHSV-TK/GCV*-loaded microbubbles-treated group, and approximately 68 ± 8 mm^3^ for the untreated group [104].

Another technique of gene delivery therapy is the RNA interference technique, which consists of protein expression inhibition. The RNA interference technique includes small interfering RNA (siRNA) and short hairpin RNA (shRNA). Zhao et al. used this technique on an orthotopic C6 glioma rat model with NGR-conjugated shBirc5-loaded liposome attached to microbubbles. *Birc5* is a protein in the family of apoptosis inhibitors. The *Birc5* gene is only expressed in malignant tumors and not in normal tissue. Thus, a plasmid containing shRNA for the *Birc5* gene could enter the cell, decrease *Birc5* gene transcription in a targeted manner, promote tumor cell apoptosis, and reduce angiogenesis without affecting normal cells. The triple function agent (tumor cell targeting, delivering gene, and BBB opening) in conjunction with FUS exhibited a significant therapeutic effect, higher than the control group: median survival times were 38 and 21 days, respectively [110].

Besides, Chen et al. have proposed another cancer treatment possibility combined with molecular targeting: des-octanoyl ghrelin-conjugated microbubbles were loaded with TGFβ1 inhibitor (LY364947). Transforming growth factor TGFβ plays an essential role in the functional regulation of tumor interstitium; it also controls the permeability of the BBB and reduces the permeability of the brain’s endothelial cell. Therefore, inhibition of TGFβ in cancer cells is expected to improve the therapeutic effects of chemotherapy. Thus, the deposition of doxorubicin in mice brain tissues was higher for the mice treated with TGFβ1 inhibitor-loaded conjugated bubbles (45 ± 5 µg/g of tissue) than for the group treated with unloaded conjugated bubbles (35 ± 5 µg/g of tissue). The median survival time was also increased (44 days for loaded microbubbles, 38 days for unloaded microbubbles) [88].

As mentioned earlier, anti-cancer drug-loaded bubbles have also been explored for cancer treatment (Figure 6) [109]. Interestingly, Fan et al. [106] proposed an alternative to boron neutron capture therapy consisting of radiotherapy based on boron agent delivery to the brain [151]. For a more efficient tumor-targeted delivery of boron, the authors fabricated boron-containing nanoparticles-loaded microbubbles for the treatment of the glioma-bearing mice model. The complex successfully disrupted the BTB and delivered boron into the brain tumor (76.6 ± 3.6% of boron uptake in tumor 4 min after drug delivery) [106].

Several studies came up with other innovative drug delivery. Briefly, Zhao et al. used phosphatidylserine nanoparticles-microbubbles complexes to monitor inflammatory reaction [113]. The complex successfully and safely opened the BBB and activated the microglia/macrophage in the rat brain. In addition, Liu et al. used sulfur nanoparticles-quercetin complex embedded in the microbubble shell for Alzheimer’s treatment [86]. Those microbubbles successfully opened the BBB on mice and allowed for a rapid accumulation of nanoparticles-quercetin complex in the brain, leading to improved Alzheimer’s disease outcome (significant increase of success on Morris water maze experiment after treatment). Finally, Ha et al. proposed a drug delivery system by binding microbubble with ultrasound-sensitizing dye-incorporating nanoparticles. They successfully delivered those nanoparticles to the U87MG (human glioblastoma cell line) located in the mouse brain (fluorescence intensity 1.5 times higher than the control group) [90].

Among the multifunctional bubbles for drug delivery strategies found in the recent literature, most of them have to be destroyed to release the active principle embedded in their shell. However, bubble destruction results in a significant risk of brain damage (hemorrhage), which is not acceptable. Although safe BBB disruption with no evidence of acute or chronic inflammation was pointed out after bubble destruction by some studies [89,105], DNA delivery has been reported without destroying the DNA-loaded bubble [104]. Indeed, DNA loaded in bubbles might penetrate cells through endocytosis when the bubble undergoes stable cavitation [104].

## 3. Discussion

### 3.1. Safety

To move forward into clinical trials, newly designed agents for FUS-induced BBB opening have to be safe. Meng et al. provide a synthesis of ultrasound parameters and drug characteristics that influence FUS’s safety profile to enhance drug delivery [27]. The safety is reachable in a particular window of parameters, including ultrasound parameters [129], microbubble dose [75,127,155], and the biocompatibility of the materials used to design the agent. The low-intensity ultrasound used for FUS-induced BBB opening is expected to have minimal thermal effects. Negligible temperature elevations (<3 °C) were reported in studies with different ultrasound parameters in rats, rabbits, and sheep [27]. Mechanical index (MI) can be used to avoid excessive cavitation of bubbles. MI is defined as the ratio between the peak-negative pressure and the square root of the center frequency (*f*_c_) [129]. As for direction, the British Medical Ultrasound Society has set a limit for diagnostic applications of ultrasound of MI = 0.7 for bubbles. For therapeutic applications, no limitations have been set yet. BBB disruption without hemorrhage has been reported with bubbles (proteins and nanoparticles self-assembled shell with C_3_F_8_ core) for MI = 0.25 on Sprague Dawley rats, among others [85]. McMahon et al. have demonstrated the bubble dose dependence of inflammatory response after BBB disruption [155]. While a 10 µL/kg dose of Definity did not show any inflammatory response on the rat brain, a 100 µL/kg dose resulted in an inflammatory response accompanied by edema, neuronal degeneration, neutrophil infiltration, and micro-hemorrhage.

### 3.2. Ongoing Challenges and Future Directions

To date, twenty-three clinical trials have been (3), are presently (4) or will be (16) conducted to settle a proof of concept of BBB opening with FUS (clinicaltrials.gov) for the treatment of several conditions: glioblastoma or brain metastases (14), Alzheimer disease (6), Parkinson disease (2) and amyotrophic lateral sclerosis (1). To the best of our knowledge, five teams have published the results from those trials in peer-reviewed journals [156,157,158,159,160,161]. These phases I and IIa clinical trials use clinically approved microbubbles for diagnostic imaging to facilitate the clinical translation of the procedure.

Agents that are not already clinically approved need to fulfill several requirements as sufficient accumulation in the target tissue, biocompatibility, clearance from the body, higher efficacy than clinically-approved microbubbles, cost-effectiveness, large scale production, easy formulation, packaging, and storage [32,128].

Characterization of cavitation thresholds (and vaporization threshold if needed), size distribution, in vivo circulation persistence, and electrical charge measurement of the newly designed agent must be performed whenever used for BBB disruption with FUS. The design of shells that can encapsulate drugs or MR contrast agents or bind with ligands for precise targeting should be further investigated as these approaches present numerous promising leads.

A more recent approach uses gas vesicles present in buoyant microorganisms to engineer theranostic agents. These gas vesicles are protein shell structures with a few hundreds of nanometers that allow coupling focused ultrasound to cells using genetic tools and improve circulation stability due to the carrier cells’ intrinsic properties [162,163,164]. This technology creates a unique opportunity for cell-based therapeutics and diagnostics. Cells can be sonoporated with unprecedented control, for example, to kill cancer cells, release a molecular payload such as drugs, or potentially disrupt the BBB at smaller capillaries in comparison with microbubbles.

## 4. Conclusions

In summary, this review describes the theranostic capability of microbubbles in noninvasive therapeutic ultrasound interventions. The therapeutic applications of microbubbles have been explored in vivo, where the effects of cavitation were linked to drug delivery enhancement in the CNS due to the temporary increase of BBB permeation. We discussed the use of both microbubbles originally designed as imaging contrast agents and microbubbles and droplets engineered specifically for therapy application with increased stability in blood circulation and penetration, as well as the development of multimodal contrast agents used for BBB disruption and dynamic contrast-enhanced MRI. The fabrication methods and agent characterization (microbubbles and droplets) were described in terms of optimal size, concentration, dissolution, clearance rate, and vaporization. We also described the agents’ use as carriers for magnetic particles, genes, neurotrophic factors, and anti-cancer drugs, which provide superior spatial control of drug release and higher concentration doses at targeted regions. Finally, the ongoing challenges involved in clinical trials revealed the importance of developing agents and methods based on cavitation monitoring to assess the safety and efficacy during brain treatment. Such developments may potentially include the use of acoustic biomolecules that offer promising results for cell-based therapeutics and diagnostics.

## Figures and Tables

**Table 1:** Composition and phase of sono-sensitive agents used for BBB opening over the past 5 years.

Commercial Name	Phase	Core	Shell	References
Definity (Lantheus Medical Imaging)	gas	C_3_F_8_	lipid	[12,13,16,36,37,38,39,40,41,42,43,44,45,46,47,48,49,50,51,52,53]
SonoVue/Lumason (Bracco)	gas	SF_6_	lipid	[15,36,37,54,55,56,57,58,59,60,61,62,63,64,65,66,67,68]
Optison (GE Healthcare)	gas	C_3_F_8_	protein	[38,69,70,71,72,73,74]
SIMB (Advanced Microbubbles Inc)	gas	“gas”	lipid	[75]
Vevo MicroMarker (Fujifilm)	gas	C_4_F_10_ and N_2_	lipid	[76]
BG8235 similar to BR-38 (Bracco)	gas	C_4_F_10_	lipid	[77]
Targeson Inc	gas	PFC	lipid	[78]
USphere (Trust Bio-sonics)	gas	C_3_F_8_	lipid	[37]
Sonazoid (GE Healthcare)	gas	C_4_F_10_	lipid	[68]
Sonazoid (GE Healthcare)	AC: gas and liquid	Sonazoid bubbles: C_4_F_10_ Homemade droplets: C_6_F_12_	lipid	[79]
Non-commercial	liquid	C_3_F_8_ or C_4_F_10_	lipid	[33]
Non-commercial	liquid	C_5_F_12_	lipid	[80]
Non-commercial	gas	C_3_F_8_	protein	[81,82,83]
Non-commercial	gas	C_3_F_8_	self-assembled polymeric nanoparticles	[84]
Non-commercial	gas	C_3_F_8_	self-assembled polymeric nanoparticles and protein	[85]
Non-commercial	gas	air	polymer	[86,87]
Non-commercial	gas	C_5_F_12_	polymer	[88]
Non-commercial	gas	SF_6_	lipid	[68,89,90]
Non-commercial	gas	C_4_F_10_	lipid	[6,11,14,39,68,91,92,93,94,95,96,97,98,99,100,101,102,103]
Non-commercial	gas	C_3_F_8_	lipid	[38,68,104,105,106,107,108,109,110,111,112,113,114,115,116,117,118,119,120,121,122,123,124,125,126]

AC: Acoustic cluster; PFC: perfluorocarbon; C_3_F_8:_ octafluoropropane; C_4_F_10_: decafluorobutane; C_5_F_12_: dodecafluoropentane; C_6_F_12_: perfluoromethylcyclopentane; SF_6_: sulphur hexafluoride; N_2_: nitrogen.

**Table 2:** Comparison of different commercial bubbles regarding BBB opening.

Ref	Bubble Type	Injection Dose (µL/kg)	Number of Bubbles per mL	Animal	Acoustic Parameters	Evans Blue Leakage	Damages Score
Bing et al. [38]	Optison	30	7 × 10^8^	Sprague Dawley rats (230–300 g)	PnP = 0.47 MPa	High	NA
	Definity	6	1 × 10^10^		*fc =* 0.75 MHz	Moderate	NA
					PRF = 1 Hz		
					duration = 120 s		
					burst = 10 s		
Shin et al. [36]	SonoVue	30	2 × 10^8^	Sprague Dawley rats (250–300 g)	PnP = 0.3 MPa	4.45%	1
					*fc =* 0.5 MHz		
	Definity	20	1 × 10^10^		PRF = 2 Hz	13.72%	0
	Definity	100	1 × 10^10^		duration = 60 s	16.35%	1
					burst = 10 s		
Wu et al. [37]	SonoVue	200	2 × 10^8^	Sprague Dawley rats (250–300 g)	PnP = 0.39 MPa	0.79 ± 0.24 µM	0
					*fc =* 0.4 MHz		
					PRF = 1 Hz		
	Definity	4	1 × 10^10^		duration = 120 s	0.52 ± 0.25 µM	0
					burst = 10 s		
	USphere	1.43	2.8 × 10^10^			0.2 ± 0.04 µM	0
Omata et al. [68]	Sonazoid	3333	9 × 10^8^	ddY mice (6 week old)	Intensity = 0.5 W/cm^2^	18 ± 7 µg/g brain	0
	SonoVue	30,000	1 × 10^8^		*fc =* 3 MHz	5 ± 1 µg/g brain	0
					PRF = 10 Hz		
					duration = 180 s		
					burst = 50 s		

PnP = Peak negative Pressure; *fc =* central frequency; PRF = Pulse repetition frequency; NA: non-assessed; the damage score grades hemorrhage and tissue damages: grade 0—normal tissue, grade 1—scattered or discontinuous erythrocyte extravasation, grade 2—continuous extravasation or microhemorrhage, grade 3—hemorrhage with necrotic damage or gross hemorrhage [27].

**Table 3:** Stability and cavitation thresholds assessment for sono-sensitive agents for BBB opening; without other specifications, cavitation thresholds are detected with in vitro passive cavitation detection. The storage stability was assessed by taking samples at several times after agent formulation and checking its echographic stability [80], cavitation emissions [108,125], concentration [39], or therapeutic effect [102].

Phase	Core	Shell	Storage Stability	In Vitro Acoustic Stability (Echography)	In Vivo Half-Life	Stable Cavitation Threshold	Inertial Cavitation Threshold	Ref.
liquid	C_5_F_12_	PEG-PLGA	stable 2 days at 4 °C	NA	NA	VT = 1.0 MPa (*fc =* 1 MHz)		[80]
liquid	C_3_F_8_ or C_4_F_10_	DSPC: DSPE-PEG2000 (molar ratio 9:1)	NA	NA	NA	C_3_F_8_ VT = 0.3 MPa		[33]
						C_4_F_10_ VT = 0.75 MPa		
						(*fc =* 1.5 MHz)		
gas	C_3_F_8_	DPPC: DSPE-PEG2000: DPTAP (molar ratio 9:2:1)	NA	relatively stable 50 min at 37 °C	NA	NA	0.3 MPa (*fc =* 1MHz)	[105]
gas	C_3_F_8_	DPTAP: DPPC: DSPE-PEG2000 (molar ratio 31,5:3,9:1,8)	NA	stable 1 h at 37 °C	10 min (male C57BL/6J mice 20–25 g)	0.3 MPa (*fc =* 1 MHz; BBB opening without damages)	0.5 MPa	[106]
							(*fc =* 1 MHz)	
gas	C_3_F_8_	DSPC: DSPE-PEG2000 (molar ratio 9:1)	NA	NA	NA	NA	0.175 MPa	[91]
							(*fc =* 0.25 MHz)	
							0.4 MPa	
							(*fc =* 1 MHz)	
gas	C_3_F_8_	DBPC: DPPA: DPPE: DSPE-PEG2000 (molar ratio 6,15:2:1:1)	NA	NA	6–8 min (Sprague Dawley rats 230–300 g)	0.21 MPa in vitro	0.59 MPa in vitro	[38]
						0.16 MPa in vivo	0.47 MPa in vivo	
						(*fc =* 0.75 MHz)		
gas	C_3_F_8_	DBPC: DPPA: DPPE: DSPE-PEG2000 (molar ratio 6:1:2:1)	stable 2 h at 5 × 10^10^ bubbles/mL	NA	10 min at 10^11^ bubbles/mL (Sprague Dawley rats 230–300 g)	0.31 MPa in vivo	0.70 MPa in vivo	[108]
						(*fc =* 0.75 MHz; 10^10^ bubbles/mL)		
gas	C_3_F_8_	DSPC: DSPE-PEG2000 (molar ratio 9:1)	NA	NA	8 min (nude mice)	NA	NA	[123]
gas	C_3_F_8_	DPPC: DPTAP: DSPE-PEG2000 (molar ratio 31,5:3,9:1,8)	NA	stable 1 h at 37 °C	NA	0.3 MPa	0.5 MPa	[107]
						(*fc =* 1 MHz)	(*fc =* 1 MHz)	
gas	C_3_F_8_	DSPC: DSPG: DSPE-PEG2000 (molar ratio 21:21:1)	NA	stable 1 h at 37 °C	7.6 min for MB 10,8 min for SPIO-DOX-MB (Sprague Dawley rats 200–250 g)	0.3 MPa	0.5 MPa	[109]
						(*fc =* 1 MHz; BBB opening without damages)	(BBB opening with damages)	
gas	C_4_F_10_	DSPC: DSPE-PEG2000 (molar ratio 9:1) or DSPC: DSPE-PEG2000-Amine (molar ratio 9:1) or DSPC: DSPE-PEG2000-Amine: DSTAP (molar ratio 7:1:2)	half-life of 2 h	NA	NA	NA	NA	[39]
gas	C_5_F_12_	PEGGM-PDSGM	NA	stable after 14 days at 37 °C	10 min (mice 25–35g)	NA	NA	[88]
gas	C_3_F_8_ or C_4_F_10_ or SF_6_	DSPC: DSPG: DSPE-PEG2000 (molar ratio 30:60:10)	NA	half-life at 37 °C	C_3_F_8_: 130 ± 50 s	NA	NA	[68]
				C_3_F_8_: 80 ± 5 s				
					C_4_F_10_: 190 ± 40 s			
				C_4_F_10_: 145 ± 35 s				
					SF_6_: 20 ± 20 s			
				SF_6_: 20 ± 5 s				
gas	C_4_F_10_	DSPC: DSPE-PEG2000 (molar ratio 9:1)	Stable 21 days	NA	NA	NA	NA	[102]
gas	C_3_F_8_	DSPC: DSPG: DSPE-PEG2000 (molar ratio 10:4:1)	relatively stable 60 min at 25 °C	NA	NA	0.3 MPa (*fc =* 1 MHz; BBB opening without damages)	0.5 MPa (BBB opening with damages)	[125]

*fc =* central frequency; NA = Non-assessed; VT: Vaporization threshold; SPIO-DOX-MB: superparamagnetic iron oxide–doxorubicin–microbubble complex; Core compositions: C_3_F_8_: octafluoropropane; C_4_F_10_: decafluorobutane; C_5_F_12_: dodecafluoropentane; C_6_F_12_: perfluoromethylcyclopentane; SF_6_: sulphur hexafluoride; Shell compositions: DBPC: 1,2-dibehenoyl-sn-glycero-3-phosphocholine; DPPA: 1,2 dipalmitoyl-sn-glycero-3-phosphate; DPPC: 1,2-dipalmitoyl-sn-glycero-3-phosphocholine; DPPE: 1,2-dipalmitoyl-sn-glycero-3-phosphoethanolamine; DPTAP: 1,2-dipalmitoyl-3-trimethylammonium-propane; DSPC: 1,2-distearoyl-sn-glycero-3-phosphocholine; DSPE-PEG2000: 1,2-distearoyl-*sn-*glycero-3-phosphoethanolamine-N-[methoxy(polyethyleneglycol)-2000]; DSPE-PEG2000-Amine: 1,2-distearoyl-sn-glycero-3-phosphoethanolamine-N-[amino(polyethyleneglycol)2000]; DSPG: 1,2-distearoyl-snglycero-3-phospho-rac-glycerol; DSTAP: 1,2-distearoyl-3-trimethylammonium-propane; PEGGM-PDSGM: poly(ethylene glycol-g-glutamate)-co-poly(distearin-g-glutamate); PEG-PLGA: poly(ethylene glycol)-poly(lactide-co-glycolic acid).

**Table 4:** Comparison of different bubbles on their stability and their abilities to open the BBB and deliver 70 kDa fluorescent dextran to the brain performed by Omata et al. [68].

Bubble Characteristics				Stability Assessments *		BBB Opening Performances			
Commercial Name	Shell	Core	Average Size (µm)	In Vitro Half-Life at 37 °C ( s)	In Vivo Half-Life ( s)	Injection (µL/kg)	Evans Blue Leakage (µg/g Brain) *		70 kDa Dextran Delivery
							*t*us *=* 0 s	*t*us *=* 3 min	
Sonazoid (GE Healthcare)	lipid	C_4_F_10_	2.11 ± 0.02	1300 ± 250	40 ± 20	3333	18 ± 7	3.2 ± 0.5	NA
SonoVue (Bracco)	lipid	SF_6_	2.23 ± 0.02	60 ± 10	20 ± 10	30,000	5 ± 1	NA	NA
NC	DSPC: DSPG: DSPE-PEG2000 (molar ratio 30:60:10)	C_3_F_8_	1.48 ± 0.02	80 ± 5	130 ± 50	1875	13 ± 3	3.5 ± 1.5	high fluorescence
NC		C_4_F_10_	1.36 ± 0.02	145 ± 35	190 ± 40	1875	10 ± 1	4.2 ± 0.2	high fluorescence
NC		SF_6_	1.63 ± 0.01	20 ± 5	20 ± 20	5000	4 ± 1	NA	weak fluorescence

NC: Non-commercial; *t*_US_: delay time of ultrasound exposure after bubble injection; C_3_F_8_: octafluoropropane; C_4_F_10_: decafluorobutane; SF_6_: sulphur hexafluoride DSPC: 1,2-distearoyl-sn-glycero-3-phosphocholine; DSPG: 1,2-distearoyl-sn-glycero-3-phosphoglycerol; DSPE-PEG2000: *N*-(carbonyl-methoxypolyethyleneglycol 2000)-1,2-distearoyl-sn-glycero-3-phosphoethanolamine; NA: Non-assessed. Acoustic parameters: Intensity = 0,5 W/cm^2^; *fc =* 3 MHz; duration = 180 s; burst = 100 s. * data obtained by graphical reading.

**Table 5:** Molecular targeting and drug/gene-complexed bubble for BBB opening and therapeutic purpose.

Ref	Core	Shell	Molecular Targeting	Drug/Gene Embedded in the Agent
[86]	air	polymer	NA	quercetin-modified sulfur nanoparticles-loaded bubble
[88]	C_5_F_12_	polymer	des-octanoyl ghrelin-conjugated bubble	TGFβ1 inhibitor (LY364947)-loaded bubble
[89]	SF_6_	lipid	NA	*GDNFp/BDNFp*-loaded liposome bound to bubble
[90]	SF_6_	lipid	NA	ultrasound-sensitizing dye-incorporating nanoparticles bound to bubble
[110]	C_3_F_8_	lipid	NGR-conjugated (targeting)/*shBirc5*-loaded (gene) liposome bound to bubble	
[104]	C_3_F_8_	lipid	anti-VEGFR2 antibody-conjugated bubble	*pLUC / pHSV-TK/GCV*-loaded bubble
[109]	C_3_F_8_	lipid	NA	SPIO-DOX-conjugated bubble
[111]	C_3_F_8_	lipid	NA	*GDNFp*-loaded cationic bubble
[112]	C_3_F_8_	lipid	NA	*pDC315*/*Nrf2*-loaded bubble
[107]	C_3_F_8_	lipid	folate-conjugated bubble	*pLUC*-loaded bubble
[105]	C_3_F_8_	lipid	NA	*pPrestin*-loaded bubble
[106]	C_3_F_8_	lipid	NA	boron-containing polyanion nanoparticles coupled with cationic bubble
[125]	C_3_F_8_	lipid	NA	PSPIO-*GDNFp*-loaded bubble
[113]	C_3_F_8_	lipid	phosphatidylserine nanoparticles-microbubble complex	

TGFβ1: transforming growth factor; *GDNFp*: a glial cell line-derived neurotrophic factor plasmid DNA; *BDNFp*: brain-derived neurotrophic factor plasmid DNA; NGR: Asn-Gly-Arg peptide; *shBirc5*: short hairpin RNA-*Birc5* gene; VEGFR2: vascular endothelial growth factor receptor 2; *pLUC*: plasmid DNA encoding luciferase gene; *pHSV-TK/GCV*: plasmid DNA encoding herpes simplex virus type 1 thymidine kinase/ganciclovir gene; SPIO-DOX: superparamagnetic iron oxide-doxorubicin complex; *pDC315/Nrf2*: plasmid DNA encoding DC315-nuclear factor E2-related factor 2; *pPrestin*: plasmid DNA encoding Prestin protein; *PSPIO-GDNFp*: superparamagnetic iron oxide coated with cationic polyethylenimine conjugated with plasmid DNA encoding glial cell line-derived neurotrophic factor; NA: Not applicable).
